# Delta Opioid Receptors Regulate Temporoammonic-Activated Feedforward Inhibition to the Mouse CA1 Hippocampus

**DOI:** 10.1371/journal.pone.0079081

**Published:** 2013-11-15

**Authors:** Xavier Rezai, Brigitte L. Kieffer, Michel J. Roux, Dominique Massotte

**Affiliations:** Department of Neurogenetics and Translational Medicine, IGBMC, Illkirch, France; UCL School of Pharmacy, United Kingdom

## Abstract

The opioid system influences learning and memory processes. However, neural mechanisms underlying the modulation of hippocampal activity by opioid receptors remain largely unknown. Here, we compared how mu and delta receptors operate within the mouse CA1 network, and used knock-in mice expressing functional delta opioid receptors fused to the green fluorescent protein (DOR-eGFP) to determine how delta opioid receptor-expressing interneurons integrate within the hippocampal circuitry. Through whole cell patch-clamp recording of CA1 pyramidal neurons from wild-type and DOR-eGFP mice, we found that mu and delta receptors both modulate spontaneous GABAergic inhibition received by these cells. Interestingly, mu but not delta receptor activation decreased the feed-forward inhibitory input evoked by Schaffer collateral stimulation. However, mu and delta agonists modulated GABAergic feed-forward inhibition when evoked upon stimulation of the temporoammonic pathway. In addition, anterograde tracing using biotinylated dextran amine injected into the entorhinal cortex of DOR-eGFP mice suggests the existence of synaptic contacts between temporoammonic afferents and delta receptor-expressing interneurons processes in CA1. Altogether, our data demonstrate a distinct modulatory role of the hippocampal network activity by mu and delta opioid receptors, and show for the first time that delta receptor-expressing interneurons in the CA1 are recruited by the temporoammonic pathway rather than the Schaffer collateral.

## Introduction

The opioid system plays a major role in the control of nociceptive pathways, modulates affective behavior [1], and is also involved in learning and memory processes [2], [3]. Mu receptors are the primary molecular target of morphine and mediate its effects on memory-related behaviors [4], [5] whereas delta opioid receptors are involved in spatial memory [6] as well as drug-context associations [7], [8] or context-induced reinstatement to drug seeking [9], [10]. All these studies point to a crucial role of mu and delta opioid receptors as modulators of hippocampal activity.

In rats, immunohistochemical [11]–[13], *in situ* hybridization [14] and electrophysiological [15], [16] data showed that mu and delta opioid receptors are mainly expressed in hippocampal GABAergic interneurons. Recently, a similar distribution was reported for murine delta receptors using DOR-eGFP knock-in mice that express delta receptors fused at their C-terminus to the green fluorescent protein (GFP) [17], [18].

From a functional point of view, opioid receptors indirectly modulate principal cell activity. In rats, application of mu or delta opioid agonists disinhibits CA1 pyramidal cells by decreasing GABA release from interneurons. However, only mu but not delta receptor activation decreases the GABAergic feed-forward inhibition activated upon Schaffer collateral (SC) stimulation on rat hippocampal slices [15]. This suggests that the neuronal populations expressing mu and delta opioid receptors are differently integrated within the CA1 network. Hence, delta receptor-expressing population could possibly be contacted by other hippocampal inputs such as the temporoammonic pathway (TA), another major entry to the CA1 that originates in the entorhinal cortical layer III [19].

Here, we combined electrophysiological recording of CA1 pyramidal cells, anterograde tracing of the TA and direct visualization of delta opioid receptor subcellular localization in DOR-eGFP knock-in mice to examine the recruitment of delta opioid receptor-expressing neurons by the SC and the TA pathways.

## Methods

### Ethics Statement

All experiments were performed in accordance with the European Communities Council Directive (26/05/2010) and approved by the local ethical committee (Com’Eth 2010-003).

### Animals

**Figure 1:**
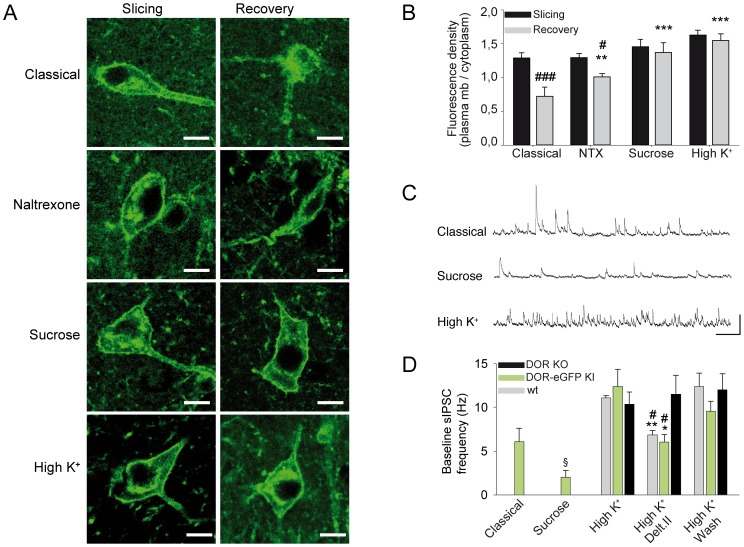
Influence of acute slice preparation protocols on receptor subcellular localization and basal interneuron activity in the CA1 area. A: Representative confocal images showing DOR-eGFP membrane localization after slicing (slicing) (left panels) and DOR-eGFP subcellular distribution following the recovery period preceding electrophysiological recordings (recovery) (right panels) in the four protocols used. Scale bars 10 µm. B: Quantification of DOR-eGFP internalization expressed as a ratio of membrane associated versus cytoplasmic fluorescence densities (n = 8–14). DOR-eGFP plasma membrane localization is preserved in the presence of sucrose 150 mM (sucrose) or upon sodium chloride substitution by potassium gluconate (High K^+^) but not in classical slice preparation protocol (classical), nor in the presence of the antagonist naltrexone 10 µM (NTX). *, p<0,05 versus baseline, ***, p<0,001 compared to classical; ^#^, p<0,05, ^###^, p<0,001 compared to slicing. C: Representative traces showing the difference in sIPSCs frequency recorded in CA1 pyramidal cell using the classical, sucrose or High K+ slice preparation protocol. Scale bar: 500 ms/50 pA. D: Histogram showing the difference of sIPSCs frequency recorded in CA1 pyramidal cell in different conditions. Frequency is significantly lower in DOR-eGFP KI mice slices prepared in the presence of 150 mM sucrose (sucrose, n = 4) than in aCSF (classical, n = 4) or upon sodium chloride substitution by potassium gluconate (K^+^ gluconate, n = 11). *, p<0.05 compared to classical. In the latter condition basal frequencies are not different in DOR-eGFP KI, wild type and DOR KO mice. Application of the delta opioid receptor agonist deltorphin II (500 nM) induces a comparable and washable decrease in DOR-eGFP KI and wild type but has no effect in DOR KO mice.

DOR-eGFP knock-in mice (n = 37) expressing the delta opioid receptor fused to GFP were generated as described previously [20] and used separately when stated or pooled with wild-type animals (n = 11). Mice deficient for the delta opioid receptor (DOR-KO, n = 5) [21] were used to verify agonist specificity in figure 1C. Animals were housed in a temperature- and humidity-controlled animal facility (21±2°C, 45±5% humidity) on a 12 h dark-light cycle with food and water *ad libitum*. Male and female mice (C57/BL6J;129svPas 50∶50%) aged 3–4 weeks (electrophysiology) or 12 weeks (tracing) were used.

### Acute Hippocampal Slice Preparation

Animals (3–4 weeks) were killed by cervical dislocation and brains quickly placed into « slicing ice-cold artificial cerebrospinal fluid (saCSF) » containing (in mM): 126 NaCl, 2.5 KCl, 2.7 MgCl_2_, 1.2 NaH_2_PO_4_, 26 NaHCO_3_, 10 glucose, 1 CaCl_2_, 0.01 MK801 for classical preparation, or 130 K-Gluconate, 14 KCl, 2 EGTA, 20 HEPES, 25 glucose, 0.01 MK801 for the potassium-gluconate protocol. Solutions were bubbled with 95%O_2_/5%CO_2_. 300 µm coronal or horizontal sections (12° angle, anterior side of the brain to the apex) were cut with a vibratome (Leica VT1000S) for experiments respectively involving SC or TA stimulation. Hippocampal slices were then incubated for 30 min at 34°C in MK801-free saCSF or 10 min for the potassium-gluconate protocol in a solution containing (in mM): 225 D-Mannitol, 2.3 KCl, 7.7 MgCl_2_, 1.25 NaH_2_PO_4_, 25 NaHCO_3_, 25 glucose, 0.5 CaCl_2_. For experiments in the presence of an opioid antagonist, naltrexone 100 mg/kg was injected intraperitoneally 30 minutes before sacrificing the animal and downstream manipulations were performed in solutions containing 10 µM naltrexone. For experiments involving sucrose, 150 mM sucrose was added in the saCSF and the NaCl concentration was only 40 mM. Before being used for electrophysiology, slices were allowed to recover at room temperature for 1 h in oxygenated aCSF containing (in mM): 126 NaCl, 3 KCl, 1.2 MgCl_2_, 1.2 NaH_2_PO_4_, 25 NaHCO_3_, 25 glucose, 1.3 CaCl_2_. All chemicals were from Sigma-Aldrich (St Louis, MO, USA) except QX-314 (Merck, Darmstadt, Germany).

### Electrophysiological Recordings

All experiments were conducted on pyramidal cells of the hippocampal CA1 region identified by their morphology. Slices were observed under infrared Nomarski optics using a 63× water immersion objective and a Hamamatsu C8484 camera mounted on a Leica DMLFSA microscope. In the patch setup, the slice was continuously perfused with bubbled aCSF at 2 mL.min^−1^ and drugs (500 nM deltorphin II, 500 nM DAGO, 2 mM Kynurenate, 50 µM CNQX and APV) were added through bath application or locally by 10 second pressure injections (picospritzer, Parker, Cleveland, USA).

Whole-cell voltage clamp recordings of membrane currents were made using patch electrodes (4–6 MΩ) pulled from GC150TF borosilicate glass capillaries (Sutter Instruments) on a horizontal puller (Zeitz Instrumente, Munich, Germany) and filled with intracellular solutions containing (in mM): 140 cesium methane-sulfonate, 10 HEPES, 1 CaCl_2_, 3 MgSO_4_, 3 ATP-Na_2_, 0.5 GTP-Na_3_, 10 EGTA, 5 QX-314, 0.07 AlexaFluor® 568 hydrazide (Molecular Probes, Saint-Aubin, France), osmolarity 318 mOsm/L, pH adjusted to 7.3 with CsOH). The junction potential (14 mV) was compensated online; series resistance (15–40 MΩ) was not compensated but monitored during the whole recording (calculated from the exponential decrease of the capacitive current induced by a 10 mV step imposed to the cell every 2 min) and only if stable, were cells kept for statistical analysis. Membrane currents were acquired using a Multiclamp 700 A amplifier, a Digidata 1322A interface and the pCLAMP9 software (Molecular Devices, Sunnyvale, CA, USA). Spontaneous and evoked inhibitory post-synaptic currents (sIPSCs and eIPSCs, respectively) were isolated by holding cells at the reversal potential of non selective cationic currents (+10 mV), while a holding potential of −70 mV, corresponding to the reversal potential of GABA-evoked current, was used to isolate evoked excitatory post-synaptic currents (eEPSCs). sIPSCs were filtered (1 kHz low-pass filter) and sampled at 2 kHz for later off-line analysis (pCLAMP). Events were automatically detected by a sliding-template algorithm, manually checked and counted to construct time plots of the sIPSCs frequencies and amplitudes. eIPSCs or eEPSCs were evoked using bipolar tungsten microelectrodes (0.5 MΩ) connected to a constant current isolated stimulator (DS3, Digitimer ltd., Hertfordshire, England) and placed either between CA1 and CA2 area in the stratum radiatum for SC stimulation, or at the subiculum level in the stratum lacunosum moleculare for TA stimulation.

### Imaging of Acute Hippocampal Slice

Slices were taken for imaging either directly after slicing (named slicing) or at the end of the recovery period just before starting recording (named recovery). Recovery period was 90 minutes (30 minutes recovery at 34°C followed by 1 hour at room temperature in a) for the classical a, sucrose and naltrexone protocols and 70 min for the high K^+^ protocol (10 min recovery in mannitol solution at 34°C followed by 1 h in aCSF at room temperature). Slices were fixed in 4% paraformaldehyde, 0.1 M phosphate buffer saline pH 7.3 (PBS), washed 3 times in PBS for 10 min and mounted in Mowiol with 4′, 6-diamidino-2-phenylindole (DAPI).

### Anterograde Tracing

12-week old mice were anesthetized with Ketamine/Xylazine (100/10 mg/kg, i.p.) and maintained in a stereotaxic frame. The skin above the skull was disinfected and opened with a single scalpel cut of 2 cm. The skull was washed with PBS and a 1 mm diameter hole was drilled in the appropriate location. 0.2 M potassium acetate containing 4% biotinylated dextran amine (BDA, Invitrogen) was unilaterally delivered into the layer III of the entorhinal cortex (stereotaxic coordinates (mm): LM +4.1; AP −3; DV +5.2) using a glass micropipette (tip diameter: 10–30 µm) and iontophoretically injected (4 µA current, 7 seconds ON/7 seconds OFF during 20 minutes, Midgard source, Stoelting). The cut was stitched up, lidocain cream was applied on the wound and animals were taken out of the stereotaxic frame and put on a 35°C plate to recover. The whole procedure typically lasted less than 45 minutes and the mice usually recovered within 15 minutes after the surgery. Animals were sacrificed 2–15 days later and perfused with 10 mL 0.1 M phosphate buffer pH 7.3 (PB) followed by 50 mL of 4% paraformaldehyde in PB. Dissected brains were post-fixed in 4% paraformaldehyde in PB overnight and cut with a vibratome (Leica VT1000S) in 60 µm slices. DOR-eGFP signal was amplified by immunochemistry as described previously [17] and BDA revealed with AlexaFluor® 594 conjugated streptavidin (1∶2000) (Molecular Probes).

### Image Acquisition and Fluorescence Quantification

Samples were observed with a confocal microscope (SP2, Leica) with a 40× oil objective and images acquired with the LCS (Leica) software. The method used for image analysis was as described in Scherrer et al. (2006). Briefly, quantification of internalization was performed using the IMAGEJ software. Nuclear fluorescence defined the background level. Cytosolic fluorescence intensity was subtracted from whole cell fluorescence intensity to obtain surface fluorescence intensity. Fluorescence intensity values were divided per surface unit (pixel) to obtain densities. Ratio of surface (Df surf) versus cytoplasmic (Df cyto) fluorescence densities was calculated to normalize data across neurons examined. A value of 1.0 results from equal densities of DOR-EGFP at the cell surface and in the cytoplasm. For each condition, four animals were used. Two slices per animal and 1–2 neurons per slice were analyzed for surface versus intracellular DOR-eGFP distribution.

### Statistical Analysis

Effect of slice preparation on DOR-eGFP localization was assessed using a two-way ANOVA and a Newman-Keuls post-hoc test. Non-parametric test were chosen instead of ANOVA for electrophysiology experiments to account for the small size of the sampling. For comparisons involving genotype effects, Mann-Whitney tests with Holm-Bonferroni corrections were performed, whereas significance of drugs effects and washout were tested with the repeated measure Friedman rank test and a Newman-Keuls post-hoc test. Latencies of eEPSC vs eIPSC were compared using paired t-test.

## Results

### Acute Slice Preparation Strongly Impacts on Delta Opioid Receptor Subcellular Localization

Using DOR-eGFP knock-in mice, we previously showed that, *in vivo,* delta opioid receptors are localized at the plasma membrane in the basal state and internalize in intracellular vesicles upon activation by an agonist [20], [22]. Imaging hippocampal slices immediately after slicing by fluorescence microscopy confirmed DOR-eGFP localization at the cell surface (Fig. 1A). However, after slice recovery in aCSF as used for patch-clamp recordings, most DOR-eGFP fluorescence was detected intracellularly (Fig. 1A&B). Different protocols were then tested to preserve delta receptor plasma membrane localization.

We first examined whether addition of the opioid antagonist naltrexone (100 mg/kg i.p. followed by 10 µM in saCSF and aCSF) would be sufficient to prevent receptor internalization. Unfortunately, this approach proved inefficient to maintain the receptor at the plasma membrane (Fig. 1A&B).

We then attempted to block delta receptor internalization via clathrin-coated pits by adding sucrose at all steps of the slice preparation [23]–[25]. Low concentration (75 mM) was inefficient (not shown) whereas high concentrations (150 mM), though preserving cell surface localization (Fig. 1A&B), deeply affected neuronal survival. Indeed, observation of the surface of the slice under Nomarski optics showed numerous swelled cells with reduced refringence, their nuclei becoming clearly visible. Moreover, recording of CA1 pyramidal cells revealed extremely low sIPSC frequency (2.0±0.8 Hz, n = 4) compared to slices prepared in classical conditions (6.1±1.5 Hz, n = 4) (Fig. 1B).

We finally tested a strategy in which the electrical activity in the slice would be temporarily decreased by substituting sodium chloride with potassium-gluconate [26]. This protocol, designed to keep cells slightly hyperpolarized when returned to normal aCSF following slice cutting, preserved DOR-eGFP cell surface localization (Fig. 1A&B) and resulted in high, stable sIPSC frequency values (10.3±1.5 Hz, n = 11) (Fig. 1C). This protocol was chosen for all subsequent experiments, as they involved DOR stimulation.

To further control our experimental conditions, basal frequencies and effect of the delta opioid receptor agonist deltorphin II were compared in wild type, DOR-eGFP and DOR knockout mice. Basal sIPSC frequencies were not significantly different between DOR-eGFP (10.3±1.5 Hz, n = 11), wild type (7.8±1.3 Hz, n = 8) and DOR knock-out mice (10.4±1.8 Hz, n = 5), indicating no effect from deletion or mutation of the delta opioid receptor on basal GABAergic inhibition received by pyramidal cells. Application of the selective delta agonist deltorphin II (500 nM) decreased the baseline sIPSC frequency by 49.5±11.7% in DOR-eGFP mice (n = 7) and by 38.4±7.0% in wild type mice (n = 4) with no modification in DOR knock-out mice (8.0±13.6%, n = 5) (Fig. 1C). Since sIPSC frequencies and response to delta opioid agonist were no significantly different between DOR-eGFP and wild-type mice, data from both genotypes were pooled in subsequent experiments.

### The Schaffer Collateral Pathway Recruits mu but not Delta Opioid Receptor-expressing Interneurons

**Figure 2:**
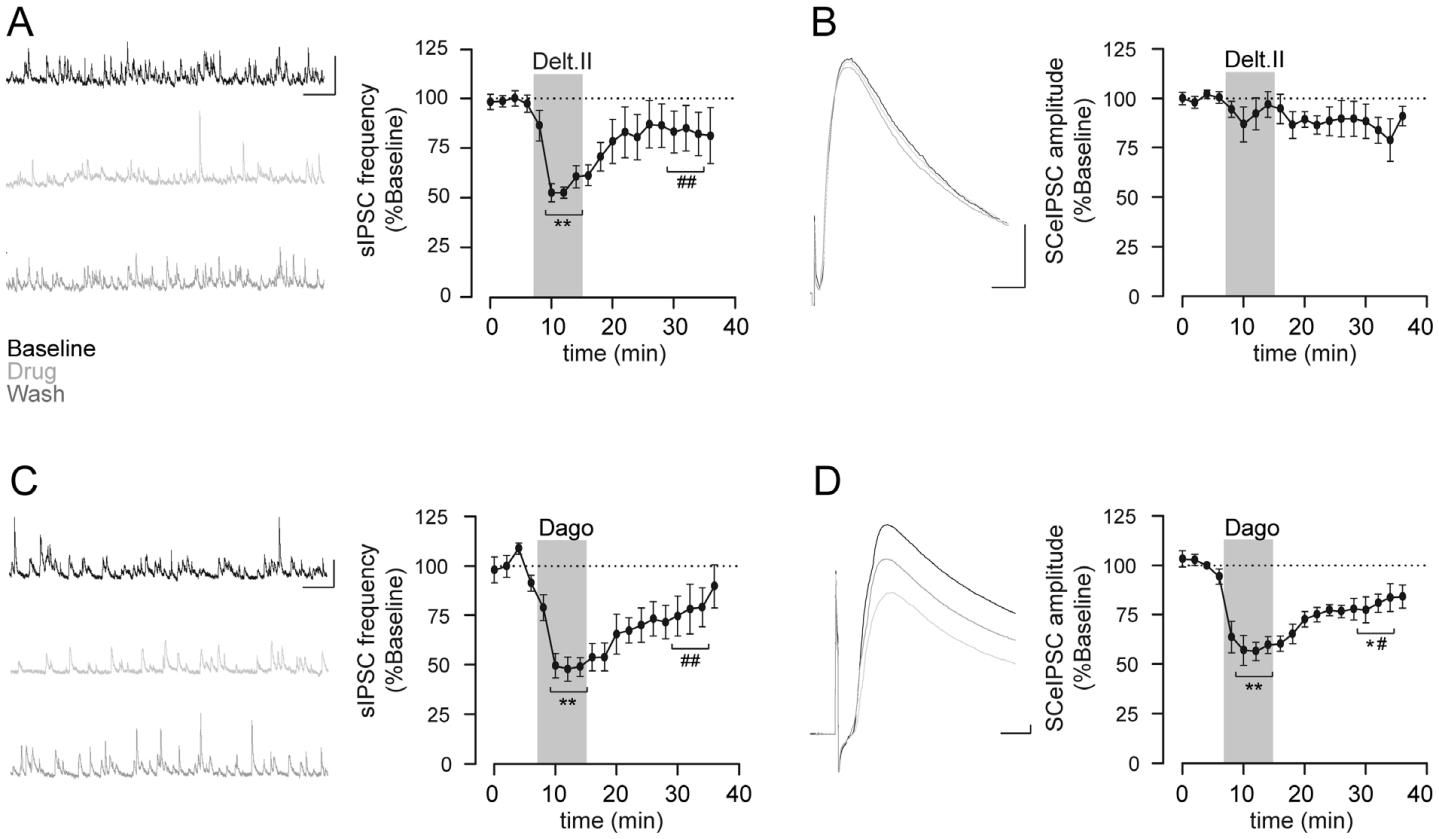
Mu but not delta opioid receptor expressing interneurons are recruited by the SC pathway. The selective delta opioid receptor agonist deltorphin II 500(A) but not SC-eIPSC amplitude (B). The selective mu agonist DAGO 500 nM decreases both sIPSC frequency (C) and SC-eIPSC amplitude (D). Representative traces and corresponding time course. * or ^#^, p<0.05, ** or ^##^, p<0.01 versus baseline or drug respectively. Scale bar 50 pA/500 ms for sIPSC and 100 pA/10 ms for SC-eIPSC.

Application of the selective mu agonist DAGO (500 nM) or delta agonist deltorphin II (500 nM) both decreased sIPSC frequencies (by 51.2±5.6% (n = 6) and 44.9±3.8% (n = 9) respectively) (Fig. 2A & 2C). DAGO and Deltorphin II (500 nM) had no significant effect on sIPSC amplitude (decreased by 21.9±8.9 (n = 6) and 17.2±7.2 (n = 9) respectively) and no effect on holding current was observed. Data are in good agreement with previously published results obtained from rats [15], [27].

We then focused on opioid modulation of interneurons recruited by the SC. Upon SC stimulation, we performed whole-cell recording of CA1 pyramidal cells held at the reversal potential of EPSC (+10 mV) or IPSC (−70 mV), resulting in the isolation of eIPSC and eEPSC, respectively. Analysis of evoked currents peak latencies revealed statistically shorter latencies for eEPSC (6.78±1.13 ms) than for eIPSC (10.73±1.85 ms, p = 0.018), as expected for mono-synaptic excitation and di-synaptic (feed-forward) inhibition.

Application of DAGO (500 nM) decreased eIPSC amplitude by 42.3±5.4% (n = 6) (Fig. 2B). However, deltorphin II (500 nM) failed to induce a significant decrease in eIPSC amplitude (8.1±7.5%, n = 9) (Fig. 2B). Our data therefore suggest that, in mice, SC afferents recruit mu but not delta opioid receptor-expressing interneurons.

### The Temporoammonic Pathway Recruits both Mu and Delta Opioid Receptor Expressing Interneurons

**Figure 3:**
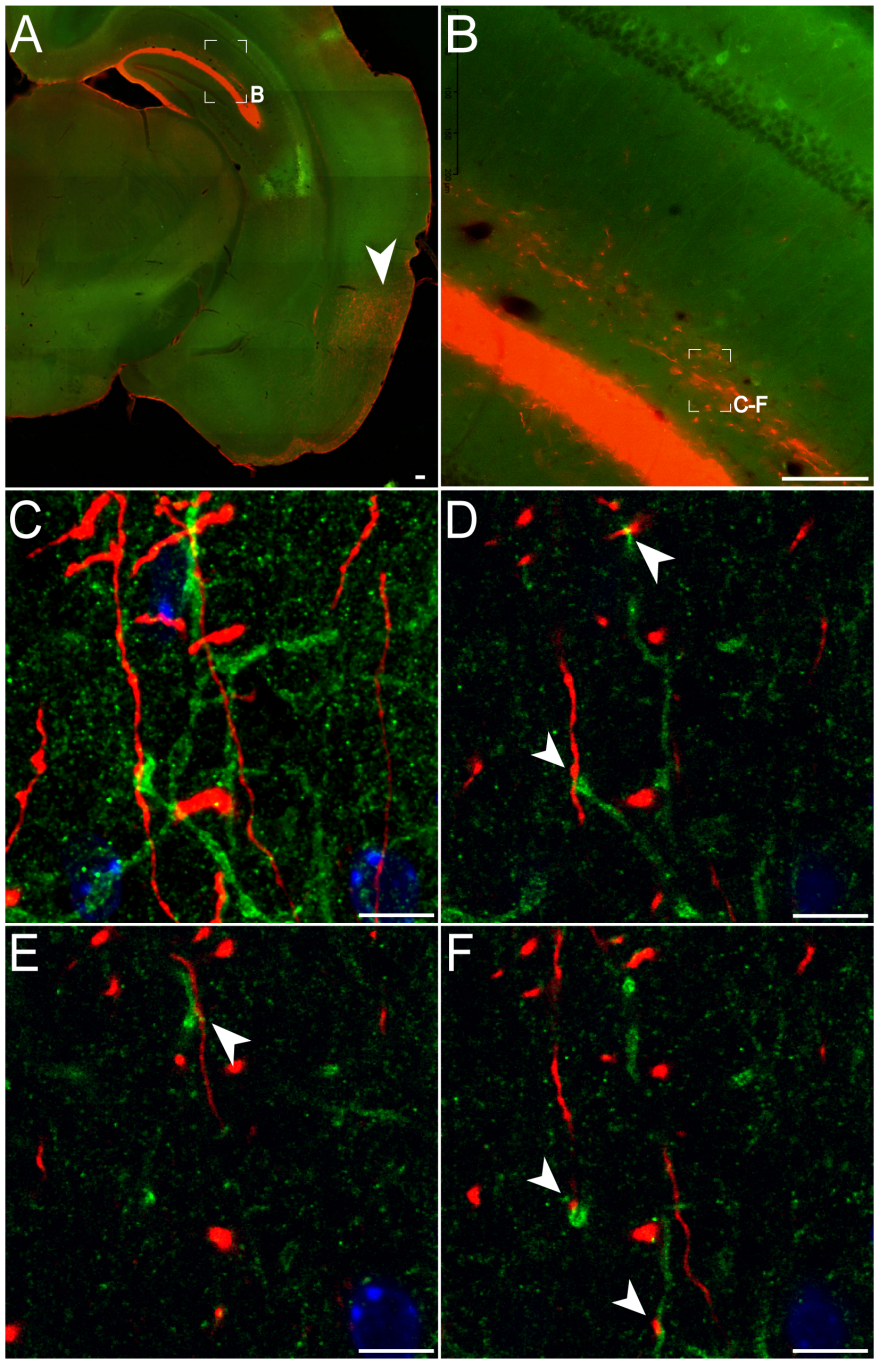
In the CA1 stratum lacunosum-moleculare, TA fibers and DOR-eGFP expressing neurites are in close vicinity. A: General view of a coronal slice showing the tracer injection point in the entorhinal cortex (arrowhead) and fibers of the perforant path. Scale bar 100 µm. B: Detail of the CA1 area delineated in A, showing TA projections (red) in the stratum lacunosum moleculare. Scale bar 100 µm. C–F: Representative 9µm thick Z projection (C) and single plan confocal images (D–F) from the zone delineated in B, showing close vicinity (arrowheads) between TA fibers (red) and DOR-eGFP expressing neurites (green) in the stratum lacunosum-moleculare. Nuclei are stained with DAPI. Scale bar 10 µm.

Recent evidence points to an implication of the TA pathway in spatial memory [28]. Also, both delta and mu opioid receptors are expressed in baskets cells that could receive TA inputs [18], [29] but no direct neuroanatomical connectivity has been demonstrated so far. We thus took advantage of the DOR-eGFP knock-in mouse and performed anterograde tracing experiments by injecting BDA in the entorhinal cortex (Fig. 3A). Observation by confocal microscopy of DOR-eGFP and BDA-associated fluorescence in the stratum lacunosum-moleculare layer of three mice (Fig. 3B) revealed that TA fibers and DOR-eGFP expressing neurites are located in close vicinity, suggesting that synaptic contacts can occur (Fig. 3C–F).

We then explored mu and delta modulation of interneurons recruited by the TA pathway. This pathway is a branch of the perforant path, entering the hippocampus via the subiculum and directly contacting CA1. We performed TA stimulation in horizontal slices, in which its integrity is better preserved than in coronal slices. Stimulating electrode was placed in the stratum lacunosum moleculare of the subiculum. Selectivity of the TA recruitment was ensured by performing two additional cuts: one between CA3 and CA1 to avoid activation of the Schaffer collateral by the classical tri-synaptic pathway and the other in the stratum radiatum between CA1 and the subiculum to prevent stimulation from directly activating CA1 interneurons. Comparison of the field potential changes evoked in the stratum lacunosum-moleculare and stratum radiatum of CA1 upon SC and TA stimulation indicated that such cuts efficiently prevented antidromic SC stimulation from contaminating the TA-evoked response (data not shown, and [30]). Whole-cell patch clamp recording of pyramidal cells after TA stimulation showed eIPSC with statistically longer peak latencies than eEPSC (14.1±3.8 ms versus 9.6±0.7 ms, p = 0.035) suggesting a di-synaptic nature of the TA eIPSC as expected, rather than a direct recruitment of interneurons by the stimulation electrode, minimized by the cut made in the stratum radiatum. This was further confirmed as inhibiting the glutamatergic transmission between the TA pathway and the interneurons by perfusion of the slice with a cocktail of glutamatergic blockers (APV & CNQX 50 µM+Kynurenate 2 mM) successfully abolished most of the evoked current (78.4±9.6% decrease, p = 0.029).

**Figure 4:**
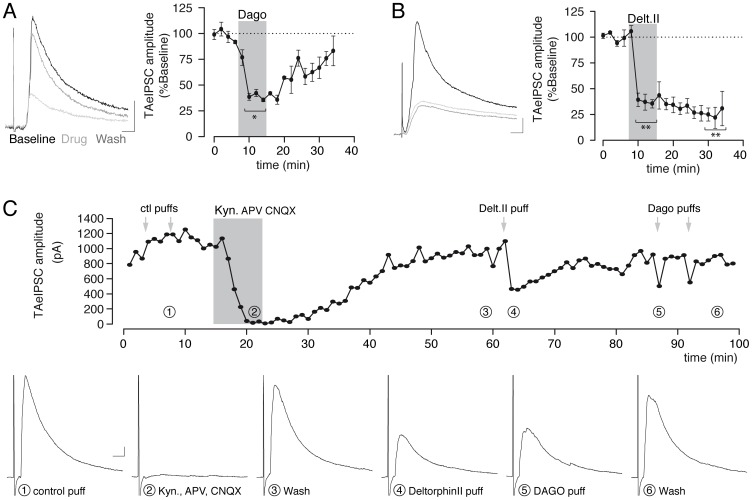
Mu and delta opioid receptor expressing interneurons are recruited by the TA pathway. TA-eIPSCs amplitude is decreased following DAGO 500 nM (A) or deltorphin II 500 nM (B) bath application in DOR-eGFP knock-in mice. *, p<0.05, **, p<0.01 versus baseline or drug respectively. (C) Representative recordings, coming from the corresponding numbers on the time-course, showing washable decrease of TAeIPSC induced by local puff application of deltorphin II (4) and DAGO 500 nM (5). In the same cell, a control aCSF puff had no effect (1) and the specificity of the temporo-ammonic pathway recruitment was demonstrated by the strong reduction of eIPSC observed in presence of a cocktail of glutamatergic blockers (2, kynurenate 2 mM, APV & CNQX 50 µM). Bath applications are indicated by gray areas, puffs by arrows. Scale bar 100 pA/10 ms.

Application of the mu selective agonist DAGO (500 nM) or the delta selective agonist deltorphin II (500 nM) both decreased eIPSC amplitude (61.3±0.4% (n = 3) and 62.8±4.6% (n = 6) respectively, Fig. 4A&B). The effect of deltorphin II on eIPSCs could not be washed. This was unlikely due to an eIPSC run-down since i) the effect of DAGO was partially reversed, and ii) a decrease in eIPSC amplitude was not observed prior to deltorphin II exposure, though we varied the time of application between 6 and 36 minutes after the start of the recording (data not shown). To minimize exposure to deltorphin II, short applications (10 s) were performed with a puff pipette next to the recorded pyramidal cell. Under these conditions, TA eIPSC amplitude was decreased by 37.6±5.2% and the effect could be fully washed out (n = 11, p<0.01). In half of the recordings, a cocktail of glutamatergic blockers was also applied, which successfully abolished the TAeIPSC, further confirming its di-synaptic nature (decrease of 83.5±7.8%). Control aCSF puff showed no significant modification TAeIPSC amplitude (decrease of 2.3±5.6%, n = 4) indicating no mechanical effect of the pressure injection. On one cell (Fig. 4C), we managed to switch the puff pipette twice during the experiment with the aCSF puff having no effect (decrease of 0.9%), while following puffs of DAMGO and deltorphin II reduced the TAeIPSC amplitude by 55.7% and 61.3%, respectively. Altogether, data suggest that both mu and delta opioid receptor expressing interneurons are recruited by the TA pathway.

## Discussion

In this study, we examined mu and delta opioid receptor modulation of both SC and TA pathways in the mouse CA1 area by a combination of electrophysiology, anterograde labeling and fluorescence confocal microscopy.

DOR-eGFP knock-in mice enable direct visualization of delta opioid receptors with subcellular localization, which led to uncover unexpected spontaneous receptor internalization during mouse hippocampal slice preparation. Receptor internalization classically occurs upon activation by an agonist [31]. In our slice preparation, internalization may result from peptide release occurring after slicing through opioidergic neurons or from ischemia leading to Ca^2+^ entry, neuron depolarization and/or unspecific neuropeptide release. We thus tested several strategies to prevent receptor activation and subsequent internalization. Slice preparation in the presence of naltrexone, a classical opioid antagonist, proved inefficient. On the other hand, the internalization process was slowed down by sucrose addition as shown previously for opioid receptors in neuroblastoma cell lines [24] or neuronal cultures [25] but neuron viability was deeply affected when slices were returned from high sucrose to aCSF. Finally, sodium chloride substitution with potassium-gluconate prevented delta opioid receptor internalization without alteration in cellular morphology or sIPSC frequency. Indeed, this protocol transiently decreases the electrical activity of the slice likely by limiting the entry of calcium and other extracellular ions into neurons thereby keeping them slightly hyperpolarized thus maximizing neuron survival [26].

Despite growing interest for the implication of opioid receptors in learning and memory processes and drug-context associations [7]–[10], few electrophysiological data are available regarding the role of delta opioid receptors within the hippocampal circuitry. Early studies by Lupica and coll. in rats observed that delta opioid receptor activation, while inhibiting spontaneous inhibition received by CA1 pyramidal cells, had no effect on SC mediated feed-forward and recurrent inhibition, contrary to mu opioid receptor activation [15], [27]. This suggested a distinct distribution of the two receptors among inhibitory interneurons in the rat hippocampal network. Here, we found comparable results in mice, indicating that the SC pathway is modulated by mu but not delta receptors in both rodent species.

Most importantly, our data demonstrate that delta receptor expressing neurons are recruited by the TA pathway. These receptors could be expressed in a population of basket cells that would not respond to SC stimulation but would selectively be activated by the TA pathway. Additional experiments are however required to unambiguously establish this point. The intrahippocampal trisynaptic loop constitutes a unidirectional route that originates in the entorhinal cortex. Mossy fibers convey information from the dentate gyrus to the CA3, then, SC fibers from the CA3 to the CA1. The TA pathway, on the other hand, directly connects the entorhinal cortex to the CA1 by projecting on pyramidal cells but also on interneurons of the stratum lacunosum-moleculare and basket cells [29]. This entry has been much studied in the context of epilepsy [32] and, more recently, was shown to play a role in long-term memory consolidation [33] and spatial representation processes [28]. Activation of the TA pathway results in a net global inhibition of the pyramidal cells preventing firing of action potentials [19]. In addition, inhibition of the SC excitatory input by the TA pathway leads to reduced CA1 cell firing [19]. Importantly, activation of mu opioid receptors drastically modulates the TA inhibition exerted on the SC input during theta rhythms [34]. Our data show for the first time that this pathway is modulated by both mu and delta opioid receptors.

The involvement of delta opioid receptors in TA rather than SC pathway is intriguing. Recent evidence has indicated that delta opioid receptors are key players in drug-context-associations, and likely facilitate this learning process (Marinelli et al., 2009, Le Merrer et al., 2011). On the other hand, requirement of the TA pathway for long-term spatial memory consolidation has been established [35]. In the context of opioid abuse, chronic exposure to morphine or heroin likely results in mu opioid receptor desensitization [36] and, as a consequence, delta receptors may become the main target for endogenous opioids in the CA1. We may speculate that increased delta receptor function and the subsequent asymmetric change in SC/TA activities could reinforce the positive perception of environmental cues associated with drug taking. The importance of delta receptors in those processes, therefore, deserves further studies, and may open novel perspectives in our understanding of hippocampal physiology and opioid regulation of learning processes.

## References

[1] Kieffer BL, Evans CJ (2009) Opioid receptors: from binding sites to visible molecules in vivo. Neuropharmacology 56 Suppl 1205–212. 18718480 10.1016/j.neuropharm.2008.07.033 PMC2950281

[2] McQuiston AR (2008) Layer selective presynaptic modulation of excitatory inputs to hippocampal cornu Ammon 1 by mu-opioid receptor activation. Neuroscience 151: 209–221. 18065149 10.1016/j.neuroscience.2007.09.077 PMC2276608

[3] Jafari-Sabet M, Jannat-Dastjerdi I (2009) Muscimol state-dependent memory: involvement of dorsal hippocampal mu-opioid receptors. Behavioural brain research 202: 5–10. 19447274 10.1016/j.bbr.2009.03.010

[4] Zhu F, Yan CX, Zhao Y, Li PP, Li SB (2011) Effects of pre-training morphine on spatial memory acquisition and retrieval in mice. Physiology & behavior 104: 754–760. 21839103 10.1016/j.physbeh.2011.07.014

[5] Olmstead MC, Franklin KB (1997) The development of a conditioned place preference to morphine: effects of microinjections into various CNS sites. Behavioral neuroscience 111: 1324–1334. 9438801 10.1037//0735-7044.111.6.1324

[6] Robles Y, Vivas-Mejia PE, Ortiz-Zuazaga HG, Felix J, Ramos X, et al (2003) Hippocampal gene expression profiling in spatial discrimination learning. Neurobiology of learning and memory 80: 80–95. 12737936 10.1016/s1074-7427(03)00025-x

[7] Shippenberg TS, Chefer VI, Thompson AC (2009) Delta-opioid receptor antagonists prevent sensitization to the conditioned rewarding effects of morphine. Biological psychiatry 65: 169–174. 18950747 10.1016/j.biopsych.2008.09.009 PMC3832215

[8] Le Merrer J, Plaza-Zabala A, Del Boca C, Matifas A, Maldonado R, et al (2011) Deletion of the delta opioid receptor gene impairs place conditioning but preserves morphine reinforcement. Biological psychiatry 69: 700–703. 21168121 10.1016/j.biopsych.2010.10.021

[9] Ciccocioppo R, Martin-Fardon R, Weiss F (2002) Effect of selective blockade of mu(1) or delta opioid receptors on reinstatement of alcohol-seeking behavior by drug-associated stimuli in rats. Neuropsychopharmacology 27: 391–399. 12225696 10.1016/S0893-133X(02)00302-0

[10] Marinelli PW, Funk D, Harding S, Li Z, Juzytsch W, et al (2009) Roles of opioid receptor subtypes in mediating alcohol-seeking induced by discrete cues and context. Eur J Neurosci 30: 671–678. 19686472 10.1111/j.1460-9568.2009.06851.x PMC2772149

[11] Drake CT, Chavkin C, Milner TA (2007) Opioid systems in the dentate gyrus. Prog Brain Res 163: 245–263. 17765723 10.1016/S0079-6123(07)63015-5

[12] Drake CT, Milner TA (2002) Mu opioid receptors are in discrete hippocampal interneuron subpopulations. Hippocampus 12: 119–136. 12000113 10.1002/hipo.1107

[13] Commons KG, Milner TA (1997) Localization of delta opioid receptor immunoreactivity in interneurons and pyramidal cells in the rat hippocampus. The Journal of comparative neurology 381: 373–387. 9133574

[14] Stumm RK, Zhou C, Schulz S, Hollt V (2004) Neuronal types expressing mu- and delta-opioid receptor mRNA in the rat hippocampal formation. The Journal of comparative neurology 469: 107–118. 14689476 10.1002/cne.10997

[15] Lupica CR, Proctor WR, Dunwiddie TV (1992) Dissociation of mu and delta opioid receptor-mediated reductions in evoked and spontaneous synaptic inhibition in the rat hippocampus in vitro. Brain research 593: 226–238. 1360320 10.1016/0006-8993(92)91312-3

[16] Zieglgansberger W, French ED, Siggins GR, Bloom FE (1979) Opioid peptides may excite hippocampal pyramidal neurons by inhibiting adjacent inhibitory interneurons. Science 205: 415–417. 451610 10.1126/science.451610

[17] Rezai X, Faget L, Bednarek E, Schwab Y, Kieffer BL, et al (2012) Mouse delta opioid receptors are located on presynaptic afferents to hippocampal pyramidal cells. Cell Mol Neurobiol 32: 509–516. 22252784 10.1007/s10571-011-9791-1 PMC5590642

[18] Erbs E, Faget L, Scherrer G, Kessler P, Hentsch D, et al (2012) Distribution of delta opioid receptor-expressing neurons in the mouse hippocampus. Neuroscience 221: 203–213. 22750239 10.1016/j.neuroscience.2012.06.023 PMC3424326

[19] Empson RM, Heinemann U (1995) The perforant path projection to hippocampal area CA1 in the rat hippocampal-entorhinal cortex combined slice. J Physiol 484 (Pt 3): 707–720. 10.1113/jphysiol.1995.sp020697 PMC1157954 7623286

[20] Scherrer G, Tryoen-Toth P, Filliol D, Matifas A, Laustriat D, et al (2006) Knockin mice expressing fluorescent delta-opioid receptors uncover G protein-coupled receptor dynamics in vivo. Proc Natl Acad Sci U S A 103: 9691–9696. 16766653 10.1073/pnas.0603359103 PMC1480468

[21] Filliol D, Ghozland S, Chluba J, Martin M, Matthes HW, et al (2000) Mice deficient for delta- and mu-opioid receptors exhibit opposing alterations of emotional responses. Nature genetics 25: 195–200. 10835636 10.1038/76061

[22] Pradhan AA, Becker JA, Scherrer G, Tryoen-Toth P, Filliol D, et al (2009) In vivo delta opioid receptor internalization controls behavioral effects of agonists. PLoS One 4: e5425. 19412545 10.1371/journal.pone.0005425 PMC2672171

[23] Heuser JE, Anderson RG (1989) Hypertonic media inhibit receptor-mediated endocytosis by blocking clathrin-coated pit formation. J Cell Biol 108: 389–400. 2563728 10.1083/jcb.108.2.389 PMC2115439

[24] Lecoq I, Marie N, Jauzac P, Allouche S (2004) Different regulation of human delta-opioid receptors by SNC-80 [(+)-4-[(alphaR)-alpha-((2S,5R)-4-allyl-2,5-dimethyl-1-piperazinyl)-3-meth oxybenzyl]-N,N-diethylbenzamide] and endogenous enkephalins. J Pharmacol Exp Ther 310: 666–677. 15102931 10.1124/jpet.103.063958

[25] Minnis JG, Patierno S, Kohlmeier SE, Brecha NC, Tonini M, et al (2003) Ligand-induced mu opioid receptor endocytosis and recycling in enteric neurons. Neuroscience 119: 33–42. 12763066 10.1016/s0306-4522(03)00135-0

[26] Dugue GP, Dumoulin A, Triller A, Dieudonne S (2005) Target-dependent use of co-released inhibitory transmitters at central synapses. J Neurosci 25: 6490–6498. 16014710 10.1523/JNEUROSCI.1500-05.2005 PMC6725433

[27] Lupica CR (1995) Delta and mu enkephalins inhibit spontaneous GABA-mediated IPSCs via a cyclic AMP-independent mechanism in the rat hippocampus. J Neurosci 15: 737–749. 7823176 10.1523/JNEUROSCI.15-01-00737.1995 PMC6578260

[28] Ito HT, Schuman EM (2011) Functional division of hippocampal area CA1 via modulatory gating of entorhinal cortical inputs. Hippocampus. 10.1002/hipo.20909 PMC3627339 21240920

[29] Kiss J, Buzsaki G, Morrow JS, Glantz SB, Leranth C (1996) Entorhinal cortical innervation of parvalbumin-containing neurons (Basket and Chandelier cells) in the rat Ammon’s horn. Hippocampus 6: 239–246. 8841824 10.1002/(SICI)1098-1063(1996)6:3<239::AID-HIPO3>3.0.CO;2-I

[30] Dvorak-Carbone H, Schuman EM (1999) Long-term depression of temporoammonic-CA1 hippocampal synaptic transmission. J Neurophysiol 81: 1036–1044. 10085331 10.1152/jn.1999.81.3.1036

[31] Cahill CM, Holdridge SV, Morinville A (2007) Trafficking of delta-opioid receptors and other G-protein-coupled receptors: implications for pain and analgesia. Trends Pharmacol Sci 28: 23–31. 17150262 10.1016/j.tips.2006.11.003

[32] Avoli M, D’Antuono M, Louvel J, Kohling R, Biagini G, et al (2002) Network and pharmacological mechanisms leading to epileptiform synchronization in the limbic system in vitro. Prog Neurobiol 68: 167–207. 12450487 10.1016/s0301-0082(02)00077-1

[33] Remondes M, Schuman EM (2002) Direct cortical input modulates plasticity and spiking in CA1 pyramidal neurons. Nature 416: 736–740. 11961555 10.1038/416736a

[34] McQuiston AR (2011) Mu opioid receptor activation normalizes temporo-ammonic pathway driven inhibition in hippocampal CA1. Neuropharmacology 60: 472–479. 21056047 10.1016/j.neuropharm.2010.10.029 PMC3014450

[35] Remondes M, Schuman EM (2004) Role for a cortical input to hippocampal area CA1 in the consolidation of a long-term memory. Nature 431: 699–703. 15470431 10.1038/nature02965

[36] Bailey CP, Connor M (2005) Opioids: cellular mechanisms of tolerance and physical dependence. Curr Opin Pharmacol 5: 60–68. 15661627 10.1016/j.coph.2004.08.012

